# The correlation between serum Cyclophilin A level and severity, prognosis of craniocerebral injury

**DOI:** 10.3389/fneur.2022.968071

**Published:** 2022-11-28

**Authors:** Peng-Fei Li, Jing-Chen Zhang, Xu-Jian He, Jian-Hua Niu, Wei-Fang Wu, Tong Li

**Affiliations:** Department of Intensive Care, The First Affiliated Hospital, Zhejiang University School of Medicine, Hangzhou, China

**Keywords:** Cyclophilin A, craniocerebral trauma, prognosis, severity, Glasgow Coma Scale (GCS) score

## Abstract

**Background:**

To investigate the value of serum Cyclophilin A(Cyp A) in evaluating the prognosis of patients with different severity of craniocerebral injury.

**Methods:**

The clinical data of patients with craniocerebral injury treated in the Department of Emergency from July 2014 to August 2017 were collected. The patients were divided into survival group and death group, good neurological function group and poor neurological function group with 28-day prognosis and were divided into mild (13–15) group, moderate (9–12) group, and severe (3–8) group with Glasgow Coma Scale (GCS) score. Clinical parameters such as Cyp A and mortality in groups and the relationship between Cyp A and GCS score were compared and its predictive value for prognosis was analyzed with Binary Logistics regression, Cox proportional hazards model and kaplan-meier survival curve.

**Results:**

In a single-center retrospective study, 503 patients were enrolled, including 365 males and 138 females; serum Cyp A in the survival group was significantly smaller than the death group [18.7 (10.1, 51.5) ng/mL vs. 149.8 (79.5, 194.4) ng/mL, *P* < 0.005]. There were significant differences in mortality and Cyp A levels between patients with different severity of craniocerebral injury (*P* < 0.001). Serum Cyp A levels were negatively correlated with GCS scores in all patients with craniocerebral injury, mild, moderate, or severe craniocerebral injury (*r* = −0.844, *r* = −0.256, *r* = −0.540, *r* = −0.531, *P* < 0.001). Predictive value of Serum Cyp A level for all patients with craniocerebral injury, mild, moderate, and severe craniocerebral injury is 0.890, 0.789, 0.806, and 0.833, respectively. Logistics regression analysis showed that lactate (*OR* = 1.260, *95%CI*: 1.023–1.551) and Cyp A (*OR* = 1.021, *95%CI*: 1.011–1.031) were positively correlated with death (*P* < 0.05), Lactic acid (*HR* 1.115; *95%CI*:1.001–1.243; *P* = 0.048), GCS score (*HR* 0.888; *95% CI*: 0.794–0.993; *P* = 0.038), Cyp A levels (*HR* 1.009; *95% CI*: 1.004–1.013; *P* < 0.001) had a significant effect on short-term mortality. Similar results were seen when neurologic function was used as the outcome. Kaplan-meier survival curve analysis found survival rate of patients with Cyp A level below the cut-off value was significantly higher.

**Conclusion:**

Serum Cyp A has a certain predictive value for the prognosis of patients with different severity of craniocerebral injury. Among them, patients with severe craniocerebral injury have the highest predictive value and mild craniocerebral injury patients have the least.

## Introduction

Craniocerebral injury is one of the most common acute and critical illnesses in emergency departments. In recent years, with the rapid development of modern construction industry and transportation industry, the number of patients with craniocerebral injury has also increased year by year, resulting in increased intracranial pressure and Decreased cerebral blood perfusion, causing ischemic necrosis of brain cells, affecting the function of the central nervous system in the brain. In severe cases, it can lead to death or disability of the injured (1). However, most of the current clinical examination methods for evaluating craniocerebral injury are limited to imaging, such as head CT, MRI, etc., which are time-consuming and labor-intensive, and the examination cost is high, which is not conducive to the early evaluation of the prognosis of the injured. Some scholars found that the secretion of Cyclophilin A(Cyp A) in brain tissue of rats with traumatic brain injury increased (2, 3). Serum Cyp A level is closely related to the disease severity and 30-day adverse neurological outcome of patients with subarachnoid hemorrhage (4), indicating that Cyp A may be an effective indicator for evaluating the prognosis of patients with craniocerebral injury. However, there are few reports on the prediction of the prognosis of patients with mild, moderate and severe craniocerebral injury by serum Cyp A levels. Therefore, this paper analyzed the clinical data of 503 patients with craniocerebral injury to explore the role of serum Cyp A levels in evaluating craniocerebral injury. The prognostic value of patients with brain injury and its relationship with the severity of brain injury aims to provide more rapid, simple and effective indicators for the assessment and treatment of patients with brain injury. The report is as follows.

## Methods

### Study design

The data of 986 injured patients admitted to the emergency department of our hospital from July 2014 to August 2017 were collected. After excluding those patients presentation with did not go to the emergency department of our hospital within 24 h after trauma (*n* = 104), with severe injury to other parts (*n* = 325), with age <18 years old (*n* = 9), with heart and brain, liver, kidney and other important organ basic diseases (*n* = 34), with use of anticoagulant drugs within 6 months before injury or with coagulation dysfunction (*n* = 11). Finally, 503 patients with craniocerebral injury were included. The study protocols were approved by the Ethics Committee of The First Affiliated Hospital, Zhejiang University School of Medicine.

### Data collection

A total of 503 patients were finally included. The gender, age, cause of trauma, Venous blood was collected from the cubital vein within 3 h after admission. Clinical severity of head trauma was assessed by Glasgow Coma Scale (GCS) score on admission by 2 experienced emergency physicians. Serum Cyp A was detected by the American BIORAD Coda automatic enzyme immunoassay analyzer. Laboratory data were obtained from the Clinical Laboratory Department of The First Affiliated Hospital, Zhejiang University School of Medicine. All patients immediately took life-saving rescue interventions, including oxygen inhalation, debridement, drug administration, tracheal intubation, tracheotomy, cerebral resuscitation, and anti-shock treatments. The prognosis of the patients was followed up by telephone after 28 days. Neurologic outcome was assessed using the Glasgow-Pittsburgh Cerebral Performance Category (CPC) and was dichotomized as either good (CPC 1 and 2) or poor (CPC 3 to 5) (5). The primary outcome was 28-day mortality. The secondary outcome was 28-day neurologic outcome.

### Statistical analysis

SPSS 22.0 statistical software was used for analysis. The Kolmogorov-Smirnov test was used to test the normality of the quantitative data. Since all the data were non-normally distributed, they were expressed as the median (quartile) M(IQR). With the 28-day prognosis as the observation end point, the 503 patients included in the study were divided into survival group (459 cases) and death group (44 cases). The differences in clinical parameters such as serum Cyp A level and GCS score were compared between the two groups by the two-sample Mann-Whitney U. According to the Glasgow Coma Scale (GCS), the patients with traumatic brain injury were divided into mild (GCS 13–15) groups, medium (GCS 9–12) groups, and severe (GCS 3–8) groups, and the mortality and serum Cyp A levels of patients were compared between the three groups by Kruskal-Wallis univariate ANOVA (k samples) test. Count data were expressed by the number of cases and percentages, and comparison between groups was performed by *X2* test. Cochran-Armitage trend test was used to compare the trend of the mortality rate in mild groups, medium groups, and severe groups. Spearman rank correlation analysis was used to explore the correlation between serum Cyp A level and GCS score and the receiver operating characteristic (ROC) curve was drawn to predict the prognosis of patients with different severity of craniocerebral injury. Based on the optimal threshold values of Cyp A, we divided craniocerebral trauma patients into Cyp A ≤ 68.9 ng/ml groups and Cyp A **>** 68.9 ng/ml groups, we plotted Kaplan–Meier survival curves by use of 28-day mortality data, and compared groups by the log-rank test, and then multiple logistic regression analyses and cox proportional hazards model analysis with a forward stepwise were conducted. The *p-*value of *P* < 0.05 was considered statistically significant.

## Results

### Clinic information of patients

A total of 503 patients were finally included, including 365 (72.6%) males and 138 (27.4%) females. The age was 49.0 (34.0, 61.0) years and there was no statistical difference in gender and cause of trauma. The GCS score of the survival group was significantly higher than that of the death group, which was 15 (14, 15) points vs. 6.5 (4.0, 11.8) points, and the serum Cyp A level was significantly lower than that of the death group, which was 18.7 (10.1, 51.5) ng/mL vs. 149.8 (79.5, 194.4) ng/mL. Similar results were observed when neurological outcome was used as the outcome (Table 1).

**Table 1:** Clinic information of the study population stratified by survival status and neurologic outcome.

**Variables**	**Survival, *N =* 459**	**Death, *N =* 44**	***X^2^* or *Z* va*lu*e**	***P***	**Good neurologic outcome, *N =* 427**	**Poor neurologic outcome, *N =* 76**	***X^2^* or *Z* value**	***P***
Female, *n* (%)	128 (27.9)	10 (22.7)	0.54	0.464	116 (27.2)	22 (28.9)	0.10	0.748
Age (years)	48.0 (32.0, 61.0)	56.5 (48.3, 67.8)	−2.92	0.003	48.0 (32.0, 61.0)	55.0 (44.0, 65.5)	−2.65	0.008
Cause of trauma [*n* (%)]			7.69	0.262			4.84	0.564
Traffic accident	239 (52.1)	21 (47.7)		218 (51.1)	42 (55.3)			
Low fall (0–0.5 m, inclusive)	90 (19.6)	9 (20.5)		85 (19.9)	14 (18.4)			
Assault	48 (10.5)	2 (4.5)		42 (9.8)	8 (10.5)			
Fall from height (more than 0.5 m)	39 (8.5)	8 (18.2)		39 (9.1)	8 (10.5)			
bruise injury caused by heavy object	14 (3.0)	1 (2.3)		14 (3.3)	1 (1.3)			
Fire	1 (0.2)	1 (2.3)		1 (0.2)	1 (1.3)			
Other	28 (6.1)	2 (4.5)		28 (6.6)	2 (2.7)			
Body temperature, °C	36.7 (36.5, 36.9)	36.9 (36.4, 37.0)	−1.63	0.102	36.7 (36.5, 36.9)	36.7 (36.4, 37.0)	−0.46	0.646
Systolic blood pressure, mmHg	134.0 (120.0, 152.0)	145.0 (131.5, 172.0)	−3.26	0.001	134.0 (121.0, 152.0)	141.0 (126.8, 164.5)	−1.99	0.046
Diastolic blood pressure, mmHg	81.0 (73.0, 89.0)	84.5 (76.0, 95.0)	−1.94	0.053	81.0 (73.0, 89.0)	84.0 (73.0, 92.0)	−1.08	0.282
Heart rate, beats/min	80.0 (71.0, 92.0)	81.0 (70.0, 95.3)	−0.38	0.705	80.0 (70.0, 92.0)	85.5 (74.0, 98.8)	−2.34	0.019
Respiratory rate, breaths/min	20.0 (18.0, 21.0)	21.0 (19.0, 24.0)	−3.26	0.001	20.0 (18.0, 21.0)	21.0 (18.3, 24.0)	−3.12	0.002
White blood cell, *10∧9/l	7.4 (4.8, 12.7)	10.0 (4.8, 15.6)	−1.36	0.174	7.4 (4.8, 12.5)	8.9 (4.8, 16.0)	−0.94	0.345
Hemoglobin, g/l	140.0 (126.0, 151.0)	135.0 (114.5, 148.5)	−1.42	0.156	140.0 (128.0, 151.0)	133.5 (107.8, 149.0)	−3.10	0.002
Platelet, *10∧9/l	187.0 (150.0, 231.0)	154.0 (116.5, 221.0)	−2.44	0.015	188.0 (153.0, 231.0)	165.5 (118.5, 223.8)	−2.48	0.013
International Normalized Ratio, INR	1.0 (1.0, 1.1)	1.1 (1.0, 1.1)	−2.10	0.036	1.0 (1.0, 1.1)	1.1 (1.0, 1.2)	−3.55	<0.001
D–dimer, ug/l FEU	5.6 (0.9, 16.4)	18.8 (5.1, 40.0)	−4.20	<0.001	5.6 (0.9, 16.2)	12.2 (1.6, 39.5)	−3.31	0.001
Activated partial thromboplastin time, s	26.6 (24.0, 29.5)	27.3 (24.8, 32.5)	−1.79	0.073	26.4 (23.9, 29.3)	28.5 (25.5, 32.7)	−4.05	<0.001
Thrombin Time, s	18.0 (17.0, 19.0)	18.5 (17.0, 19.9)	−1.30	0.193	18.1 (17.0, 19.0)	18.2 (16.8, 19.6)	−0.68	0.499
Fibrinogen, g/l	2.3 (1.9, 2.8)	2.0 (1.7, 2.5)	−2.68	0.007	2.3 (1.9, 2.8)	2.1 (1.7, 2.8)	−1.94	0.053
pH	7.4 (7.4, 7.5)	7.4 (7.4, 7.4)	−1.45	0.147	7.4 (7.4, 7.4)	7.4 (7.4, 7.4)	−0.88	0.382
Lactic acid, mmol/l	1.8 (1.0, 2.6)	3.5 (2.5, 4.7)	−6.88	<0.001	1.8 (1.0, 2.6)	2.7 (2.0, 3.9)	−5.60	<0.001
C-reactive protein, mg/l	0.8 (0.5, 2.6)	0.9 (0.5, 4.6)	−0.64	0.520	0.8 (0.5, 2.5)	1.1 (0.5, 5.3)	−1.13	0.260
Na^+^, mmol/l	138.5 (136.0, 141.3)	139.0 (136.2, 141.5)	−0.05	0.958	138.6 (136.0, 141.3)	138.6 (134.3, 141.8)	−0.51	0.608
K^+^, mmol/l	3.5 (3.3, 3.8)	3.4 (3.2, 3.6)	−2.28	0.022	3.5 (3.3, 3.8)	3.4 (3.2, 3.6)	−2.57	0.010
GCS	15.0 (14.0, 15.0)	6.5 (4.0, 11.8)	−8.60	<0.001	15.0 (15.0, 14.0)	6.5 (4.0, 14.0)	−9.36	<0.001
Cyp A, ng/ml	18.7 (10.1, 51.5)	149.8 (79.5, 194.4)	−8.56	<0.001	16.4 (9.4, 46.1)	140.2 (69.5, 191.6)	−10.44	<0.001

### Comparison of mortality and Cyp A in patients with different severity of craniocerebral injury

There were significant differences in the mortality and Cyp A levels among patients with different severity of craniocerebral injury (*P* < 0.001). And the Cochran-Armitage trend test showed that with the severity of craniocerebral injury, the mortality of patients had an increasing trend (*P*_*trend*_ < 0.001), (see Table 2).

**Table 2:** Comparison of mortality and Cyp A levels in patients with different severity of craniocerebral injury.

**Craniocerebral trauma patients**	**Number of cases**	**Fatality rate (%)**	**Cyp A, ng/mL**
Mild injury	384	2.6 (10/384)	14.8 (9.0,33.1)
Moderate injury	32	21.9 (7/32)	74.6 (59.6,103.6)
Severe injury	87	31.0 (27/87)	147.2 (97.2,190.5)
*X^2^* or *H* value		*X^2^ =* 79.20	*H* = 241.60
*p*		*P* < 0.001 *P_*trend*_* < 0.001	*P* < 0.001

### Correlation between serum Cyp A value and GCS score

**Figure 1:**
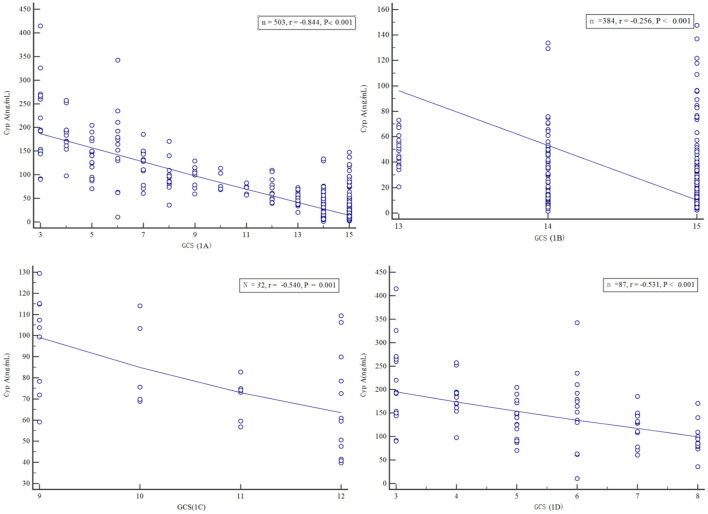
Scatter plot of serum Cyclophilin A level versus Glasgow Coma Scale score, in all craniocerebral trauma patients **(A)**, in mild craniocerebral trauma patients **(B)**, in moderate craniocerebral trauma patients **(C)**, in severe craniocerebral trauma patients **(D)**.

Spearman rank correlation analysis showed that the serum Cyp A level of all patients with craniocerebral injury was negatively correlated with GCS score (*r* = −0.844, *P* < 0.001). Based on GCS score, all patients were divided into three groups: mild (GCS 13–15) groups, medium (GCS 9–12) groups, and severe (GCS 3–8) groups and then serum Cyp A values of above three groups were closely related to the GCS score respectively (*r* = −0.256, *r* = −0.540, *r* = −0.531, *P* < 0.001). The more severe the injury in patients with craniocerebral trauma, the higher the correlation between serum Cyp A level and GCS score (Figure 1).

### Predictive value of serum Cyp A level on prognosis of patients with craniocerebral injury

**Figure 2:**
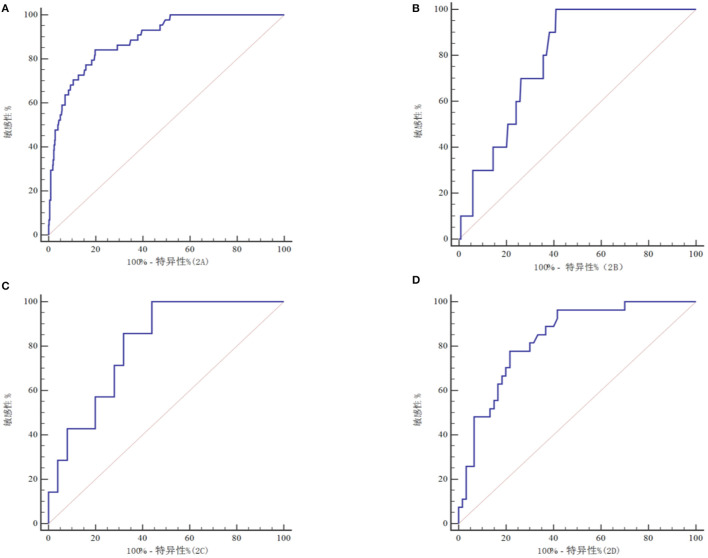
ROC curve of to predict prognosis in all craniocerebral trauma patients **(A)**, in mild craniocerebral trauma patients **(B)**, in moderate craniocerebral trauma patients **(C)**, in severe craniocerebral trauma patients **(D)**.

The ROC curve analysis showed that the serum Cyp A level had a certain predictive value for the mortality of all patients with craniocerebral injury, mild craniocerebral injury, moderate craniocerebral injury, and severe craniocerebral injury (Figure 2), which were 0.890, 0.789, 0.806, and 0.833 respectively (Table 3). The more severe the injury in patients with craniocerebral trauma, the greater the predictive value of serum Cyp A levels.

**Table 3:** Predictive efficiency of serum Cyclophilin A level for craniocerebral trauma patients' prognosis.

**Craniocerebral trauma patients**	**AUC**	**95% CI**	**Optimal threshold values, ng/mL**	**Sensitivity (%)**	**Specificity (%)**
All	0.890	0.860–0.916	68.9	84.1	80.2
Mild injury	0.789	0.744–0.829	17.2	100.0	59.1
Moderate injury	0.806	0.628–0.924	72.6	100.0	56.0
Severe injury	0.833	0.738–0.905	154.2	77.8	78.3

### Logistics regression analysis to predict 28-day mortality and poor neurologic outcome

According to the above results, the indicators with statistical significance in the prognosis of patients with craniocerebral trauma were screened. Binary Logistics regression analysis showed that lactate (*OR* = 1.260, *95%CI*: 1.023–1.551) and Cyp A (*OR* = 1.021, *95%CI*: 1.011–1.031) were positively correlated with death (*P* < 0.05), as shown in Table 4. Based on the prognosis of neurological function, D-dimer (*OR* = 0.97, *95%CI*: 0.941–0.999) correlated negatively with prognosis, while CypA (*OR* = 1.030, *95%CI*: 1.021–1.040) correlated positively (*P* < 0.05), (see Table 5).

**Table 4:** Binary logistic regression analysis of the risk factors for death of craniocerebral traumatic patients.

**Variables**	***B***	**Odds ratio**	**95%CI**	***P***
Lactic acid	0.231	1.260	1.023–1.551	0.030
Cyp A	0.020	1.021	1.011–1.031	<0.001

**Table 5:** Binary logistic regression analysis of the risk factors with poor neurologic outcome.

**Variables**	***B***	**Odds ratio**	**95%CI**	***P***
D-dimer	−0.031	0.97	0.941–0.999	0.044
Cyp A	0.030	1.030	1.021–1.040	<0.001

### Cox proportional hazards model analysis to predict 28-day mortality

The statistically significant prognostic indicators of patients with craniocerebral trauma were included in the Cox proportional hazards model for analysis, and the results showed that lactic acid (*HR* 1.115; *95%CI*: 1.001–1.243; *P* = 0.048), GCS score (*HR* 0.888; *95% CI*: 0.794–0.993; *P* = 0.038), Cyp A levels (*HR* 1.009; *95% CI*: 1.004–1.013; *P* < 0.001) had a significant effect on short-term mortality in patients with craniocerebral trauma, as shown in Table 6.

**Table 6:** Multivariable cox proportional hazard regression analysis of the risk factors for death of craniocerebral traumatic patients.

**Variables**	***B***	**HR**	**95%CI**	***P***
Lactic acid	0.109	1.115	1.001–1.243	0.048
GCS	−0.119	0.888	0.794–0.993	0.038
Cyp A	0.009	1.009	1.004–1.013	<0.001

### Kaplan-meier plot showing survival in craniocerebral trauma patients grouped by Cyp A levels

**Figure 3:**
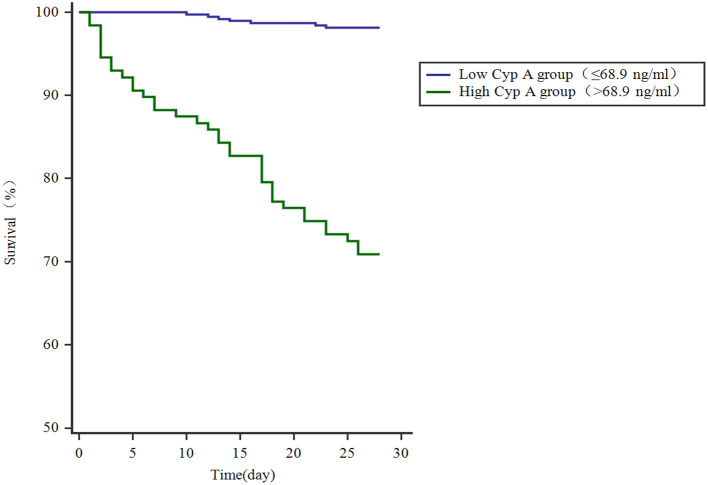
Kaplan-meier plot showing survival in craniocerebral trauma patients grouped by Cyp A levels.

We further compared 28-day mortality risk for craniocerebral trauma patients based on the cutoff value of Cyp A. 37 of 128 craniocerebral trauma patients with high Cyp A(>68.9 ng/ml) died and 368 of 375 patients with low Cyp A survived. Kaplan-meier survival curve analysis was performed and the mortality rate of patients with different groups of Cyp A was compared. The study found that the survival rate of patients with Cyp A level below the cut-off value was significantly higher (*P* < 0.001) (Figure 3).

## Discussion

Craniocerebral injury is relatively common in clinical practice. The injured often suffer from intracranial hypertension, cerebral edema, and even brain herniation. Severe cases can lead to many complications, poor prognosis and death, which seriously affects the subsequent quality of life of the injured. Therefore, Early diagnosis, timely and accurate assessment of injury severity, and effective control interventions are critical to improving prognosis.

Cyp A is a small-molecule soluble protein with conservative structure and wide distribution. It has prolyl cis-trans isomerase function and cytokine-like activity. It is involved in the regulation of immune function, inflammation, apoptosis, viral infection, cholesterol metabolism, and injury, protein repair, the occurrence and development of malignant tumors (6, 7). In the case of ischemia, hypoxia, inflammation, the body will stimulate the tissue to produce and release Cyp A to increase (8). In inflammatory diseases such as chronic obstructive pulmonary disease, lung cancer, coronary artery disease, chronic kidney disease, acute myocardial infarction, acute intracerebral hemorrhage, the serum Cyp A concentration is significantly increased (9–14). A prospective study by Jin et al. (15) included 105 patients with severe traumatic brain injury (GCS 3–8) and 105 healthy subjects, and found that serum Cyp A levels were often elevated in patients with severe traumatic brain injury, and its level was negatively correlated with GCS scores(*r* = −0.562, *P* < 0.001), which was an independent risk factor for death and poor prognosis (Glasgow Outcome Scale score of 1–3) after 90-day follow-up. However, the research objects of serum Cyp A predicting the prognosis of patients with craniocerebral injury are only patients with severe craniocerebral injury, and there are very few research reports on the prognosis of patients with mild and moderate craniocerebral injury. In addition, it is worth noting that it is unknown whether this conclusion applies to all patients with traumatic brain injury. Therefore, all patients with craniocerebral injury were included in our study, which found that serum Cyp A can effectively evaluate the prognosis of all patients with craniocerebral injury. Serum Cyp A can accurately assess the prognosis of patients with different degrees of craniocerebral injury. The more severe the injury in patients with craniocerebral trauma, the greater the predictive value of serum Cyp A levels. Thus, serum Cyp A can be used as a reliable predictor of death in patients with traumatic brain injury. In addition, the correlation analysis of this study found that the relation between serum Cyp A levels and GCS scores in patients with severe craniocerebral injury was the largest, followed by medium cases and the least in mild cases, which indicated that the more severe the injury, the lower the GCS score, the greater the serum Cyp A, and the greater the risk of poor prognosis.

It is worth noting that this study has certain limitations: Firstly, this study only included the yellow race, whether this conclusion is applicable to other populations still needs further related research. Secondly, since the venous blood collected from the cubital vein of patients with craniocerebral injury within 3 h after admission to the hospital for serum Cyp A testing, its accuracy will also be disturbed to a certain extent.

## Conclusions

Serum Cyp A is an important indicator for evaluating the severity of injury and the risk of death in patients with craniocerebral injury. The increase of Cyp A in peripheral blood is closely related to the severity and clinical prognosis of patients. The detection of this indicator can help physicians to more comprehensively assess the severity of the patient's injury and clinical prognosis, and provide physicians with the basis for medical decision-making and treatment guidance at an early stage, which has important clinical significance for reducing the patient's disability rate and improving the patient's prognosis.

## Data availability statement

The original contributions presented in the study are included in the article/supplementary material, further inquiries can be directed to the corresponding author/s.

## Ethics statement

The study protocols were approved by the Ethics Committee of The First Affiliated Hospital, Zhejiang University School of Medicine. The patients/participants provided their written informed consent to participate in this study.

## Author contributions

All authors listed have made a substantial, direct, and intellectual contribution to the work and approved it for publication.

## Conflict of interest

The authors declare that the research was conducted in the absence of any commercial or financial relationships that could be construed as a potential conflict of interest.

## Publisher's note

All claims expressed in this article are solely those of the authors and do not necessarily represent those of their affiliated organizations, or those of the publisher, the editors and the reviewers. Any product that may be evaluated in this article, or claim that may be made by its manufacturer, is not guaranteed or endorsed by the publisher.
